# Illegal Trade in Exotic Animals and Its Impacts in Slovenia—A Case Study

**DOI:** 10.3390/ani13081375

**Published:** 2023-04-17

**Authors:** Miha Dvojmoč, Valentina Kubale

**Affiliations:** 1 Policing and Security Studies, Faculty of Criminal Justice and Security, University of Maribor, Kotnikova 8, 1000 Ljubljana, Slovenia; 2 Veterinary Faculty, University of Ljubljana, Gerbičeva 60, 1000 Ljubljana, Slovenia

**Keywords:** illegal, trade in exotic pets, animals, prevention, conservation, animal welfare

## Abstract

**Simple Summary:**

Illegal trade in endangered animals poses a threat to wildlife, livestock, and pets. It also has consequences for environmental degradation, loss of biodiversity, invasive species, the spread of various diseases, and the policing of green crime. The objective of this article is to examine the illegal trade in endangered species in Slovenia, and, through structured interviews with experts and representatives of leading institutions and companies, to assess the gap between the theory and practice of effective management of the trade in wildlife. The study was conducted prior to changes in the Schengen borders that affect Slovenia and therefore assessed the status of wildlife trade regulation at that time. The study argues that, although illegal trade in endangered wildlife species was not particularly widespread in Slovenia, it is important to recognize, investigate, address, and raise awareness of the problems it could create and of the impacts the Schengen border changes were likely to have.

**Abstract:**

Currently, the illegal wildlife trade is one of the most profitable illegal enterprises in the world. The aim of our study was to determine the situation with respect to wildlife trade in Slovenia, which is mainly a transit country, before changes to the Schengen borders came into effect. The volume of trade is significant but not extensive. The most common endangered species involved in illegal trade in Slovenia are the brown bear, the peregrine falcon, the date mussel, the lady’s slipper orchid, the common snowdrop, the cyclamen, the sea turtle, the otter, and various reptile species. The smuggling of shells (date shells), ivory (ivory products), certain plants, and various hunting trophies (bears, big cats) has decreased in recent years. Nevertheless, counteracting crimes continues to be important for the conservation of some species in Slovenia, notably the lynx, and for the reduction of poaching. Improvements are needed in the detection and prevention of wildlife crime, especially in light of changes made to the Schengen borders and the consequent inclusion of new trading partners for Slovenia. The lack of people properly trained to identify, detect, and investigate wildlife crime is especially acute.

## 1. Introduction

A comprehensive understanding of deviant behavior in contemporary society requires an examination not only of interpersonal deviance but also of deviance that impacts the environment. This is one of the reasons that green and environmental criminology has developed so much in the previous decade. They are seen as an increasingly important component of national and international security policy [1]. Crime has existed since time immemorial, but it is changing as a result of globalization, which has made the wildlife trade one of the most profitable illegal enterprises in the world [2]. Although it is very difficult to measure the scale of the exotic and wildlife trade exactly, because it largely operates through informal networks, it is estimated to be worth about USD 20 billion annually [3].

As the illegal wildlife trade in endangered species spreads and expands [4], it has serious implications for environmental degradation, biodiversity loss, invasive species, and the spread of various diseases. 

The demand for endangered species and other wildlife is primarily concentrated in the affluent regions of the world where buyers can pay high prices for wildlife “commodities”. This includes the European Union, North America, Japan, and China [4,5]. The primary source countries are in Africa, Asia, and Latin America. 

In the case of Slovenia, where the exact extent of the illegal trade in wildlife is still not well understood, it nonetheless seems reasonably clear that Slovenia functions primarily as a transit state in the context of the overall global trade in animals. We, therefore, wanted to ask, with help of people involved in the animal trade and its regulation in Slovenia, what is necessary to manage wildlife trade effectively in a transit country, anticipating, more specifically, the changes that will be made for trading purposes to the Schengen borders. We also wanted to assess the impact of Slovenia’s transit trade on animal welfare. 

## 2. Illegal Trade in Animals

Not all wildlife trade is illegal and some of the goods that the wildlife trade provides are both ubiquitous and environmentally benign. This is true for a variety of wood products, for the domestic enjoyment of at least some exotic plants and animals, and for a variety of animal parts that end up in medicines and cosmetics and as parts of clothing, footwear, and handbags [6]. 

However, from a global security and veterinary perspective, illegal wildlife trafficking (IWT) can be a major cause of biodiversity loss and can pose a threat to human health. 

### Illegal Trade in Animals in Slovenia

Slovenia plays a transit role in the illegal trade of animals, mainly for partial products, but it is not a major transit route for live animal smuggling. A few cases have been detected [7]. In a period of 18 years (2000–2018), the Society for Bird Observation and Research in Slovenia (DOPPS) recorded 52 cases in Slovenia in which birds were being illegally kept and traded [8]. In 16 cases, protected bird species were sold illegally. Additionally, in the same period, there were 50 cases of illegal transport of birds through Slovenia, involving a total of 20,400 specimens. The protected birds were caught in Southeast European countries and shipped via Slovenia to customers in Latin America [7]. 

In both Slovenia and Croatia, the date mussel (*Lithophaga lithophaga*) is collected and sold on the coast at an intensity that adversely affects the entire marine ecosystem. The trade is driven by caterers and their clientele, although this protected shellfish has also appeared on the menu at various government events [9]. 

In 2017 and 2018, the Financial Administration of the Republic of Slovenia dealt with cases involving the seizure of 2486 g of caviar, a wolf-hunting trophy destined for the Netherlands, and 2100 illegally caught and frozen birds destined for Italian restaurants [9].

Slovenia is a signatory of CITES (Convention on International Trade in Endangered Species of Wild Fauna and Flora) and Endangered species listed on the so-called Red List are dealt with in Slovenia by the Protected Wildlife Species Regulation [10], which provides added protection for most endangered species. Article 344 of the Slovenian Criminal Code further provides that “Whoever unlawfully possesses, seizes, damages, kills, exports, imports, or trades in protected wild animal or plant species, protected animals or plants, or parts thereof, or products made therefrom, shall be punished by imprisonment for up to three years,” with the penalty being six months to five years if the offender commits the act in a criminal organization or if the species involved is of great or exceptional importance for the conservation of nature (Criminal Code [KZ1-UPB2]) [11].

The illegal wildlife trade is associated with various risks to wildlife, society, and finance. The attendant biodiversity risks are associated with vertebrate extinction, ecosystem alteration, genetic pollution, and the emergence of pathogens. These risks are well-known. Less well-known are the animal welfare risks caused by the suffering of animals when, for example, the animal trading process causes pain or stress, which, in turn, can affect immune systems and lead to disease outbreaks. 

On 1 January 2023, the Republic of Croatia (a neighboring country to the Republic of Slovenia) joined the Schengen area, and customs controls at the borders between Croatia and other Schengen member states, including Slovenia, were abolished. The Republic of Bulgaria and the Republic of Romania were denied accession to the Schengen area by European Union interior ministers on the same date. The Schengen area is the area in Europe where borders between countries have been abolished for EU trading purposes. It is the largest zone of free trade movement in the world and the central pillar of European integration and cooperation. The expansion of the Schengen area means that Slovenia will be able to exercise less control over the movement of animals into and through the free trade area than it does now, even if, as seems likely, transit traffic involving animals and other goods increases.

This research was conducted just before the Schengen borders were revised at the beginning of 2023. We wanted to establish the status of the animal trade prior to that date, firstly, because the existing situation is not well understood and secondly, because of the potential for animal trade to have larger adverse impacts after the Schengen changes came into effect. We designed a study that draws on structured interviews with relevant experts. We particularly wanted to examine the dimensions of the gap that separates the actual practice of regulation in the animal trade from what would ideally be required to manage that trade effectively in Slovenia, including its impacts on animal welfare.

## 3. Materials and Methods

We developed an interview questionnaire to be administered to various experts in Slovenia who are familiar with the animal trade and its impacts. After first obtaining the informed consent of respondents, data were collected between September and November of 2022. Some interviews were conducted in person, some by telephone, and some by email. Respondents were chosen because they were acknowledged by their employers as leading representatives of organizations involved either in the animal trade or its regulation.

The semi-structured interviews explored six themes that a literature review showed to be important (Table 1). The questions were open-ended and defined a subject for discussion that the interviewer could follow up on with additional questions. The in-person and telephone interviews lasted, on average, for about 45 min, with the longest lasting 1 h and 15 min. 

There were twelve respondents in all, each one directly or indirectly involved in the trade of exotic and endangered species or its regulation. The interviewees were guaranteed anonymity and confidentiality and, for data aggregation purposes, were numbered in random order. The value and relevance of their responses can be broadly gauged from a list of the institutions with which they were affiliated: the Exotic Animal Lovers Society; the Faculty of Criminal Justice and Security at the University of Maribor; the Financial Administration of the Republic of Slovenia; Pošta Slovenije, the postal service provider in Slovenia handling more than 90% of all postal deliveries; the Port of Koper, the only port in Slovenia; Slovenian Railways; the Magical Beasts Association, interested in exotic animals; the Slovenian Police; Reptiles Nest, a large and diverse animal breeder; the Slovenian Ministry of Environmental and Spatial Planning; the Veterinary and Plant Protection Section of the Administration for Food Safety; and the Slovenian Hunters Association. One additional potential respondent declined to be interviewed on the grounds that Fraport Slovenia, the manager of the only international airport in Slovenia had no relevant tasks or responsibilities. The interview responses were analyzed thematically, using the methodology described by Braun and Clarke [12,13]. All interview transcripts are available as Supplementary Material (Tables S1–S6).

## 4. Results

In this section, the information provided by respondents is briefly summarized. A more detailed picture of the responses is provided in tabular form in the Supplementary Material. 

### 4.1. Extent of Animal Trade in Slovenia

The first question asked about the extent of the animal trade in Slovenia and the way it has changed in the last ten years. The respondent from the only large exotic animal society in Slovenia said they did not detect any black-market trade. The university respondent said that Slovenia is mainly a transit country for the trafficking of protected animals, plants, and products. The respondent from the Financial Administration of Slovenia provided the trade data for the last five years, which are shown in Table 2. Seized goods included caviar from protected sturgeon species, birds (often with removed feathers, claws, and heads), live crustaceans, and hunting trophies taken from wolves and bears in the Western Balkans. Between 2012 and 2021, twenty criminal complaints were filed either by the Customs Authorities or the Financial Administration, alleging an offense under Article 344 of the Criminal Code (KZ-1). In one 2018 case involving seized birds, 1349 specimens of protected birds were found in the luggage of three Romanian nationals on a bus trip. The respondent representing the postal service said that no trafficking in exotic or endangered species had been detected in the previous ten years. The largest exotic animal breeder in Slovenia is apparently also unaware of illegal trading, although it has wide experience in the buying and selling of animals, mainly reptiles, at fairs and in the online market. The ministry respondent acknowledged contact with the customs authority and the police, who carry out seizures, as well as with the Environmental Inspectorate at the Slovenian Institute for Nature Protection, but said that the Ministry’s main role is the collection of data about seizures and the annual reporting of them to the EU and the CITES Secretariat. The reports show, based on seized specimens, that the volume of illegal trade in species protected by CITES fluctuates from year to year but has decreased over time. In the previous three years (2019–2021), customs officers have handled only 12 cases of CITES violations at border crossings. The Veterinary and Plant Protection Section of the Food Safety Administration also reports no major detections of illegal trade in endangered wildlife species, but rather isolated cases, some involving iguanas. The information provided by the respondent from the Slovenian Hunters Association about the current extent of illegal hunting in Slovenia can be found in (Tables S1–S6). 

### 4.2. The Most Common Endangered Animal Species in Slovenia

The second interview question asked about the wild and, especially, endangered species most often traded illegally in Slovenia, about the ratio of non-native to native (Slovenian) specimens in this trade, and about the species that respondents most frequently encounter in their work. The university respondent said that the species most in demand in Slovenia are the brown bear, the peregrine falcon, the date mussel, the lady’s slipper orchid, the snowdrop, the cyclamen, the sea turtle, and the otter. Offenders come from different groups. The brown bear, for example, is important to Slovenian hunters. Date shells and some bird species are considered to be a delicacy in neighboring Italy, and offenders are often caught at the border. Snake skins, crocodile products (for handbags and boots), ivory, tusks, and food supplements and medicines appeal to a much broader audience, even though their use to make such products is an environmental crime.

The respondent from the Financial Administration reported that, in those cases where illegal trade was detected by officers from the mobile units of the tax administration, Slovenia was not a destination country. The animals involved were not typically live animals and were passing through Slovenia on their way to somewhere else. The offenses were essentially violations of required paperwork, such as a failure to comply with permit conditions for the movement of protected species. 

The respondent from the postal service said that the animals most frequently encountered were those highly sought after by collectors or were parts of animals thought to have medicinal value. The respondent from the exotic animal lovers’ society, Magical Beasts, reported that Greek tortoises (*Testudo hermanni*) are still illegally collected from the wild in the Mediterranean and then frequently traded in Slovenia, more than four-fifths of them without permits or proper documentation.

The police respondent said that the illegal trade in protected animals most often involves birds and that when the trade occurs online the birds are alive. The police have also encountered the smuggling of shellfish (date shells), ivory (ivory products), trophies of various animals (bears, big cats), and the buying and selling of plants (hellebore, snowdrop, and Clusius gentian, or the flower of the sweet lady). The view of the largest breeders in Slovenia, however, is that illegal trading in native and non-native species is unlikely to amount to a significant problem because almost all species can be bought as offspring on the open market, where prices are not high, except for individual transactions in very rare and special non-native species that are too few to constitute a trade.

The ministry respondent said that in data reported over a five-year period, illegal transactions have implicated a wide range of animals; intercepted shipments in 2021 included shark teeth, leather goods, and crocodile skins, for example, and in 2020, shipments involved 37 specimens including alligator skin products, stony corals, cans of caviar, and medicinal preparations containing extracts from parts of protected plant species. In 2019, seizures involved conch shells, corals, snake wine, crocodile skin products, medicinal preparations, and caviar. In most of these cases, the destination country was Slovenia, except for the caviar, for which Slovenia was a transit country. The movements were detected mostly by examining the luggage of travelers and items shipped through the mail. 

### 4.3. Current Problem of Illegal Trade 

The third interview question tried to probe the extent of the illegal trade in wildlife, especially endangered species, in Slovenia.

The university respondent argued that wildlife crimes are not widespread in Slovenia and ought not to be considered problematic, except perhaps for their impacts on animal health. Neither of the respondents from groups representing people interested in exotic animals thought that the viability of native species was threatened by illegal trade. 

The respondents from the Financial Administration and Pošta Slovenije thought that illegal trade in protected wildlife had decreased in the last five years. They estimated that the volume of such trades was not significant now, although they also expressed the view that it was important for Slovenian authorities to keep paying attention to this type of crime. The police respondent also said that it should not be neglected, although illegal wildlife trading was not now a serious problem in Slovenia. The number of reported wildlife crimes shows a small increase every year. Furthermore, whether there is a clear pattern in the nature and frequency of cases remains an open question. 

Slovenia is clearly a transit country through which animals, mainly birds or parts of them, arrive from other countries (Romania, Bosnia and Herzegovina, Croatia) and then pass through, mainly to Western European countries (especially Italy). The police discover most cases of illegality related to birds during the control and inspection of vehicles at the roadside when the perpetrators lack the appropriate permits from the relevant authorities, regardless of whether the animals involved are protected species or not. 

### 4.4. Legal Regulation of IWT in Slovenia

The fourth interview question asked about the legal regulation of the animal trade in Slovenia and whether changes are needed at the legislative or regulatory levels.

The university respondent expressed the view that existing legislation is adequate, but that its implementation and enforcement were problematic. The postal service respondent observed that while the basic legal framework for regulating the animal trade is clear, changes in implementation were needed, especially in relation to detection and prevention.

The Financial Administration respondent also thought that the legal framework for regulating the animal trade was adequate. The respondent from Slovenian Railways agreed with this assessment but also pointed out that a broadly acceptable regulatory framework needs to be fleshed out in detail, as all the railways had done was spell out the rules that apply to the transportation of pets on passenger trains. 

The ministry respondent took the view that trading in endangered species in Slovenia was reasonably well regulated as a matter of law. Problems arose, however, with the implementation of the laws, especially when the trading of wildlife occurred through online platforms. 

The respondent from a hunting organization pointed out that, although it has people well qualified to monitor and evaluate the implementation of the laws on hunting, it has no authority to evaluate the management of wildlife in the context of broader conservation policy.

### 4.5. Competencies of the Experts in the Field

The fifth interview question asked what capabilities are needed to identify, detect, and investigate the illegal trade in wildlife, and, especially, endangered species in Slovenia, and how to determine whether changes are needed.

The university respondent said that there is a shortage of professionals in this field and that they are not sufficiently trained. This was also the view of postal service providers in Slovenia, who argued that they do not have enough equipment to detect and deal with offenses. The respondent from the Financial Administration thought that staff expertise was sufficient to monitor the implementation of existing regulations. The Port of Koper argued that its security personnel were well-trained to detect illegal wildlife trade and then forward cases to the Customs Administration for further processing.

In contrast, the Society for Magical Animals felt that there is a lack of knowledge and appropriate professionals in this area, that legislation is not effectively applied, and that there is insufficient knowledge at various levels about how to deal with exotic animals.

The police respondent emphasized that police are not experts at identifying and protecting endangered animal and plant species in the field. The police do try to coordinate and liaise with other relevant agencies, and they do occasionally organize training courses for police officers and criminal investigators on this topic. In case of a report or suspicion of illegal acts related to protected animals and plants, the police examine all information and take all necessary steps and measures based on the law and the profession.

The Ministry respondent observed that an inter-ministerial working group, which includes representatives of the police, customs, and nature protection, organizes regular training sessions for people working on wildlife crime. There remains, however, a lack of knowledge, understanding, and cooperation in the Slovenian judiciary and at the EU level. This may be mitigated by the EU Action Plan against illegal wildlife trade and by the Slovenian National Program to Prevent and Combat Crime 2019–2023, which calls for better cooperation with judicial authorities. The professional competence of official veterinarians to deal with wildlife matters less than that of those who deal with pets, and the Inspection Act and the General Administrative Procedure Act in Slovenia severely restrict the role veterinary officers can play in dealing with illegal wildlife trade. 

From the perspective of the hunting community, hunters are educated on how to prevent illegal hunting and illegal trafficking as part of the training they are required to undergo to obtain a hunting license. There are currently over 20,000 hunters in Slovenia, more than 850 of whom are registered as game wardens. 

### 4.6. Raising Awareness of Professional Competencies and Animal Welfare

The sixth interview question asked about the extent to which the Slovenian population at large is aware of the competence of professionals to identify, detect, and investigate wildlife offenses and, in particular, trade in endangered wildlife species in Slovenia and its impacts on animal welfare. 

The consensus among all respondents was that public awareness of illegal wildlife trading in Slovenia has increased in recent years, partly because of the activities respondents and their organizations have undertaken. More remains to be done, however, to bring trained personnel and funding to bear on the problem.

## 5. Discussion and Conclusions

The purpose of this study was to determine the current status of animal trafficking in Slovenia prior to changes in the Schengen borders and to ascertain whether relevant organizations in and out of government can improve their operations, as well as public perceptions of how effectively the transit trade in wildlife is being managed. In answering these questions, it is important to remember that Slovenia is a small country strategically located next to several other Balkan countries. Slovenia can be traversed by road within two to three hours after crossing the Schengen border, which makes the detection and interception of illegal wildlife trade difficult. Moreover, there are easy connections from Slovenia between east and west and from north to south in Europe. It is also important to recall that the management of wild animals in Slovenia is the responsibility of the Slovenian Hunting Association, a voluntary organization financed chiefly from its own resources and with limited help from the government in enforcing state regulations governing the taking of individual game species.

Our research confirms that Slovenia is primarily a transit country where the extent of illegal wildlife trade, though difficult to measure exactly, is clearly influenced by factors that affect the demand for and supply of wildlife products on a global basis. It is also difficult to estimate how much trade in animals goes undetected every year in Slovenia. Regardless, the expert consensus is that, in recent years, the volume of illegal trade has been decreasing. This reinforces the perception, evident in the responses to our interview questions, that neither the illegal wildlife trade nor its effects on animal welfare are matters of high priority in Slovenia. This does not mean, however, that effective interventions are unnecessary, particularly in the case of species such as the lynx, where the prosecution of crimes may well be critical for the conservation and continued survival of the species. 

Although our respondents thought that existing legislation for the management of endangered species in trade was adequate, we think there is a case to be made for adopting so-called positive lists for regulating the pet trade in Slovenia. The standard approach is to restrict or prohibit trade in exotic species when it can be shown that their survival in the wild or their welfare is endangered by allowing them to be used as pets. This is known as negative listing. The alternative, known as positive listing, permits the sale of animals as pets when a government agency has determined, using scientifically valid criteria, that it is reasonable to keep purchased animals as pets and that this does not pose a disproportionate risk to either human health or the environment [14]. This seems to us to be a sensible application of the precautionary principle. Furthermore, it is encouraging that Slovenia has the adoption and implementation of positive lists under consideration. 

Given the changes to the Schengen borders, it will be important to conduct a similar survey in a few years. Perhaps similar surveys could be conducted in Croatia as well. At this point, we would also like to highlight the difference between the countries, which is that Slovenia is small and can be transversed within two to three hours after crossing what used to be a Schengen border. This is important for the illegal trafficking of animals within Balkan countries because it makes detection and intervention so much more difficult. Slovenia clearly also has a strategic location in the Balkans and Eastern Europe and is attractive as a transit country with easy access to other European countries both on an East–West and a North–South axis. It will, therefore, remain a country through which a lot of animal trade will pass, some of it illegally. 

## Figures and Tables

**Table 1:** Semi-structured interview questions.

Question Number	Interview Question
Q1	First of all, please estimate the extent of the animal trade in Slovenia and describe, on the basis of your experience, how it has developed in the last ten years.
Q2	What species of wildlife, especially endangered species, are most often illegally traded in Slovenia? What are the most common species you encounter in your work? What is the ratio of non-native to native (Slovenian) specimens in this trade?
Q3	How problematic do you think illegal trade in wildlife is in Slovenia, especially that involving endangered wildlife species?
Q4	How do you assess the regulation of illegal trade of wild animals and especially endangered wildlife species in Slovenia? Are changes to existing laws and regulations needed?
Q5	How do you assess the competence of experts in your field to identify, detect, and investigate the illegal trade in wildlife, and especially endangered wildlife species, in Slovenia? Are changes needed in this area, and if so, what are they?
Q6	How do you assess the general level of awareness among Slovenians of the competence of experts in your field to identify, detect, and investigate the illegal trade in wildlife, especially endangered wildlife, and its impacts on animal welfare? Are changes needed in this area and if so, what are they?

**Table 2:** The extent of animal trade in Slovenia in a 5-year period.

	2017 ^(1)^	2018 ^(2)^	2019 ^(3)^	2020 ^(4)^	2021 ^(5)^
Number of seizures (CITES)	11	9	5	3	2
Number of animal species specimens (CITES)	116	46	15	12	2
Number of specimens of other protected species (birds)	/	2148	250	/	18

Description of specimens listed in the table above, by year: ^(1)^ ointment containing cobra, crocodile skin wallet, caviar, small pieces of ostrich and crocodile skin, taxidermized falcon, and crocodile skin watchband. ^(2)^ Ointment containing cobra, caviar, and bird feathers of parrots, owls, birds of prey, and corals. ^(3)^ Crocodile skin wallet, snake in wine, corals, seashells, caviar. ^(4)^ Prepared head of crocodile, corals, and caviar. ^(5)^ Crocodile parts (wallet) and shark parts (tooth).

## Data Availability

Data is contained within the article or Supplementary Material.

## Supplementary Materials

The following supporting information can be downloaded at: https://www.mdpi.com/article/10.3390/ani13081375/s1, Table S1: Q1, First of all, please estimate the extent of this trade in Slovenia, and in your experience, how has this trade developed in Slovenia in the last ten years? Table S2: Q2, What species of wildlife, especially endangered species, are most often illegally traded in Slovenia? What is the ratio of non-native to native (Slovenian) specimens in this trade? What are the most common cases you encounter in your work? Table S3: Q3, How problematic do you consider the extent of illegal trade in wildlife and especially endangered wildlife species in Slovenia? Table S4: Q4, How do you assess the legal regulation of the field of illegal trade in wild and especially endangered wildlife species in Slovenia? Are changes needed on legislative or regulatory level? Table S5: Q5, How do you assess the competence of experts in your field to identify, detect and investigate illegal trade in wildlife and especially endangered wildlife species in Slovenia? Are changes needed in this area, and if so, what are they? Table S6: Q6, How do you assess the level of awareness among the Slovenian population regarding the competence of experts in your field to identify, detect and investigate illegal trade in wildlife and especially endangered wildlife species in Slovenia and animal welfare? Are changes needed in this area and if so, what are they?
