# Supplementary material for: Illegal Trade in Exotic Animals and Its Impacts in Slovenia—A Case Study
Source: Animals (Basel). 2023 Apr 17;13(8):1375. doi: 10.3390/ani13081375 (PMC10135352; doi:10.3390/ani13081375)
Supplement: Supplementary file 1 [file animals-13-01375-s001.zip › animals-2277789-supplementary.pdf]

Table S1:

Q1	**First of all, please estimate the extent of this trade in Slovenia, and in your experience, how has this trade developed in Slovenia in the last ten years?**
**Interviewee 1**	Since we have not detected any black market trade, it is difficult to estimate the extent of this trade.
**Interviewee 2**	As far as environmental crime is concerned, Slovenia is primarily a transit country, and this also applies to trade in protected animal and plant species. Animals and plants or products that correspond to the current trend come to Slovenia from abroad.
**Interviewee 3**	The data for the last 5 years are shown in Table 2. Regarding to the problem in question, we would like to highlight some points. The seized goods regularly include caviar of protected sturgeon species, and hunting trophies of big game (wolf, bear) are smuggled into the EU from the Western Balkan countries. In addition, perpetrators remove feathers, heads and claws from killed birds to prevent professional identification and subsequent prosecution. In 2012–2021, 20 criminal charges were filed by the Customs or Financial Administration of the Republic of Slovenia on suspicion of an offence under Article 344 of the Criminal Code (KZ-1).<br>In one of the cases of seized birds in 2018 (there were three cases in total in 2018), in which officers of the mobile unit of the Financial Administration of the Republic of Slovenia seized 1,349 specimens of protected birds seized, which were found in the luggage of three Romanian nationals travelling by bus, the regional prosecutor dismissed the criminal charges on suspicion of criminal handling of protected animals and plants on the grounds that it was not proven with the necessary certainty that the suspects knew what they were transporting and that they were aware of the illegality of their actions. We also had a case of non-protected, non-native species (discovery of 68 live crustaceans by mobile unit officers) that, if released into the wild, could endanger nature and violate conservation regulations.
**Interviewee 4**	In our work over the past decade, we have not encountered or detected trafficking in exotic or endangered species. We assume that in most cases smugglers are not engaged in illegal trade, such as shipping animals and their parts through the postal network, because the likelihood of being detected is relatively high.
**Interviewee 5**	The respondent did not provide an answer.
**Interviewee 6**	The respondent did not provide an answer.
**Interviewee 7**	I guess that such trade is present because Slovenia is a transit country, but I cannot estimate the extent.
**Interviewee 8**	The respondent did not provide an answer.
**Interviewee 9**	I personally do not know of a dissatisfied trade, I myself deal with reptiles and there are enough of them legally raised in the European Union and outside it, so my view is that there are no illegal wild animals on the Slovenian market.
**Interviewee 10**	First of all, we should point out that the Ministry, as an administrative authority, is not directly responsible for the seizures, but cooperates within an inter-ministerial working group with the Customs Authority and the Police, which carry out the seizures, as well as with the Environmental Inspectorate and the Expert Panel (Institute of the Republic of Slovenia for Nature Protection). The Ministry of Environment collects the data and submits them in annual reports on seizures to EC and the Secretariat CITES. Compared to growing global trends, in Slovenia, data on the volume of illegal trade in seized specimens protected by the CITES Convention in Slovenia fluctuate from year to year, but show a decreasing trend in recent years. In the last three years (2019–2021), customs officers have handled 12 cases of CITES violations at border crossings.
**Interviewee 11**	In the last ten years, there have been no major detections of illegal trade in endangered wildlife species, or we do not have accurate data to identify, detect, and investigate illegal trade in wildlife, including exotic pet species. There are isolated cases. Several years ago, we investigated trafficking of a non-native species (iguanas). In the past, trade in servals has also been uncovered.
**Interviewee 12**	It is difficult to discuss the extent of trafficking in Slovenia from the perspective of the Slovenian Hunters' Association, as we do not keep such data and are only indirectly confronted with this problem In this context, we could talk in a little more detail about illegal hunting, which we still encounter in Slovenia. Anyone can commit the crime of illegal hunting, including hunters.

Table S2:

Q2	**What species of wildlife, especially endangered species, are most often illegally traded in Slovenia? What is the ratio of non-native to native (Slovenian) specimens in this trade? What are the most common cases you encounter in your work?**
**Interviewee 1**	The respondent did not provide an answer.
**Interviewee 2**	The most wanted species in Slovenia are traditionally the brown bear, the peregrine falcon, the date mussel, the lady's slipper orchid, the snowdrop, the cyclamen, the sea turtle and the otter. The perpetrators come from different groups; for example, one pays for a brown bear, which is usually hunted by hunters, while date shells and some birds are a delicacy in neighbouring Italy and the perpetrators are often caught with their prey at the border. One question that has long bothered me is whether the olm is also on the list of desirable species for environmental crime. In my opinion, imports from abroad predominate, often snakeskin and crocodile products (handbags, boots) and ivory or tusks (jewellery, statues, etc.), which also include dietary supplements and pharmaceuticals. As an eco-criminologist, I study organized ecological crime as a form of "ecological crime.
**Interviewee 3**	The cases handled in recent years by the officials of the Financial Administration involve specimens listed under CITES or Council Regulation (EC) No. 338/97 on the protection of species of wild fauna and flora (CITES specimens), as well as specimens of species protected under the Bern Convention (Convention on the Conservation of European Wildlife and Natural Habitats) or under national regulations on protected species (mainly birds). CITES specimens of species that did not in comply with legislation or were imported without the required CITES permits were discovered in recent years mainly in postal consignments from third countries, and the recipients of the goods were mostly natural persons. The mentioned birds were discovered during inspections by the officers of the mobile units of the Tax Administration, and according to the collected data, Slovenia is not the destination country in these cases. In the illegal bird transports leading from the Balkans to Italy, Slovenia acts as a transit country. We have not handled any cases involving live specimens.
**Interviewee 4**	We estimate that the most common animals found are those that are highly sought after by collectors, or parts of animals that are considered to have medicinal value."
**Interviewee 5**	The respondent did not provide an answer.
**Interviewee 6**	The respondent did not provide an answer.
**Interviewee 7**	Greek tortoises *Testudo hermanni*. They are still illegally collected from the wild in the Mediterranean. In Slovenia they are bred without a permit and about 80% of the tortoise population does not have proper papers.
**Interviewee 8**	Among the protected species that are most often traded illegally in Slovenia, we find mainly (dead) birds, while live birds and reptiles dominate online trade. In the past, we have handled cases of smuggling of shellfish (date shells), ivory (ivory products), trophies of various animals (bears, big cats) and certain plants (hellebore, snowdrop, Clusius gentian or the flower of the sweet lady).
**Interviewee 9**	I think lovers of reptiles, amphibians and similar species ... In Slovenia, native and non-native species are not traded illegally because almost all species can be bought as offspring on the free market and one does not even have to face confiscations and fines for illegal trade; moreover, native Slovenian species do not fetch high prices on the market as perhaps rare special non-native species do, and for this reason alone they are not interesting for illegal trade.
**Interviewee 10**	In 2021, we handled two shipments that contained parts of protected species: shark teeth and leather goods and crocodile skins. In 2020, customs officers seized five illegal shipments containing 37 specimens, namely: alligator skin products, stony corals, caviar cans, and medicinal preparations containing extracts from parts of protected plant species. In 2019, 15 specimens were seized in seven cases: conch shells, corals, snake wine, crocodile skin products, medicinal preparations and caviar. In most cases, the country of destination was Slovenia, with the exception of caviar, for which Slovenia was a transit country. The shipments were mostly sent by mail or were in the luggage of travellers. The seized items were confiscated from the perpetrators, most of whom were unknown persons.
**Interviewee 11**	At the Jože Pučnik Airport Ljubljana border crossing point, import controls for endangered animal and plant species (CITES) and controls on the movement of domestic animals other than birds for other than commercial purposes are carried out by the Ministry of Finance, the Financial Administration of the Republic of Slovenia, the Ljubljana Tax Office, the Customs Department and the Customs Clearance Department at Jože Pučnik Airport Ljubljana. Very few live animals are imported from third countries through the border crossing at Jože Pučnik Airport Ljubljana. In 2019, 2020 and 2021, we only processed cases of aquarium fish, dogs and cats of common breeds, lemurs (exchange between two zoos), one consignment of live turtles destined for a registered breeder in Slovenia and one non-commercial movement of a parrot. To our knowledge, all of these animals were imported into Slovenia. None of the animals were stopped at the border for non-compliance with endangered species conditions. Four animals were

	brought into Slovenia. Servals are considered less endangered species and do not need to be accompanied by documentation CITES (IUCN Red List). We recently handled a case where a mail shipment contained a spider. The most common animals involved in illegal trade are most likely to be feral cats, exotic birds, and exotic reptiles.
**Interviewee 12**	Slovenian hunters are part of the European Federation for Hunting and Nature Conservation (FACE), to which one million hunters belong, and they hunt the fewest species. The Game and Hunting Law (ZDLov-2004 with amendments) specifies certain mammal and bird species that are hunted as game. A government decree defines these species. In Slovenia, hunters hunt only 23 species, while in France and Italy, for example, they hunt 58 or more species. Of these species, Slovenian hunters hunt only five bird species (crow, magpie, Eurasian jay, mallard and pheasant) as "game." Other bird species that are not hunted in Slovenia are hunted in neighboring countries (heather cock, Eurasian woodcock, etc.). Exotic species in Slovenia include large carnivores, such as brown bear, wolf and lynx. Brown bears and wolves are hunted by hunters on the basis of a decree issued by the competent ministry (Ministry of Environment), but only in very limited numbers. The lynx was practically exterminated in the 1920s, partly due to illegal hunting and the peculiarity of its genetics (inbreeding). In recent years, the Slovenian Hunters' Association has helped to reintroduce the lynx to Slovenia.

Table S3:

Q3	**How problematic do you consider the extent of illegal trade in wildlife and especially endangered wildlife species in Slovenia?**
**Interviewee 1**	We have no experience with such trade. Only turtles in the Littoral region (on the Slovenian coast) and Mediterranean domestic geckos are often mistaken for escaped pets and re-released.
**Interviewee 2**	Compared to other forms of crime and organized crime, this form of crime, although not so widespread, should still be considered problematic as it affects the the survival of certain animal and plant species. Take the case of the lynx in Slovenia, which is known to often end up as a trophy with hunters, when they should be among the actors protecting it.
**Interviewee 3**	The number of cases of illegal CITES specimens has decreased in the last five years, so we do not consider the volume of illegal trade in this area to be problematic."
**Interviewee 4**	We estimate that the volume of this type of trafficking in Slovenia is currently not significant, but the question is whether the border authorities and other authorities in the Republic of Slovenia pay enough attention to this type of crime.
**Interviewee 5**	The respondent did not provide an answer.
**Interviewee 6**	The respondent did not provide an answer.
**Interviewee 7**	Native wildlife species are extremely rarely illegally declared or kept as exotic species. Every other year someone mentions that they had a smooth snake in their terrarium. And many families get some spawn in their aquarium in the spring.
**Interviewee 8**	Statistically, this trade is not a big burden in Slovenia, but dealing with crimes related to protected animal and plant species is of utmost importance, as individual species or their existence are fundamental for the preservation of nature. In Slovenia, between 10 and 15 criminal cases are processed annually for illegal handling of protected species of wild animals and plants, but the number of reported crimes is slowly increasing every year. Despite the individual cases, there is no particular pattern/nature of occurrence or time period in which multiple crimes occur. We note that Slovenia is a transit country where animals (mainly birds) or parts of them come from other countries (Romania, Bosnia and Herzegovina, Croatia) and travel through Slovenia mainly to Western European countries (Italy). The Police discover most cases of illegality related to birds during the control and inspection of vehicles (at the roadside) where the import or export of animals is not authorized, as the perpetrators usually do not have the appropriate permits from the competent authorities, regardless of whether they are protected species or other animals (exotic species – mainly trophies or parts of trophies, birds – including parrots, turtles or dogs). However, every year the police also deals with individual cases of illegal practices related to birds (hunting, keeping, possession) in Slovenia. NGOs and individuals inform us about such cases.
**Interviewee 9**	With the species I have experience with, I do not think it is a problem.
**Interviewee 10**	The police have reported poaching in recent years, with various bird species and cave animals (setting traps in caves in Slovenia) being the most common. We do not know if and how these species are involved in trafficking. However, in the past (mainly between 2002 and 2006), a large number of wild bird species were seized, destined for the Italian market through Slovenia. A successful and high-profile police operation and cross-border cooperation with Croatian authorities resulted in the seizure of date shells (smuggled from Croatia to Italy and Slovenia) a few years ago. After Slovenia's accession to the EU, and especially after Croatia's accession, when Slovenia no longer had direct external EU borders, the number of seizures decreased, as we now have only two direct ways of entering Slovenia, through the port of Koper and Jože Pučnik Airport Ljubljana, or through mail. In recent years, seized specimens have entered Slovenia mainly by mail. Any illegal trade is problematic because it affects natural populations. This is especially harmful for endangered species, as biodiversity decreases, which in addition to the natural threat factors, burdens these species both directly and indirectly. One such case occurred several years ago when an unknown perpetrator released the invasive, non-native freshwater crayfish – the marbled crayfish – into the wild. The breeding and sale of this species is prohibited.
**Interviewee 11**	In general, we believe this type of trade is more common than we observe. The scale is problematic because there is limited ability to monitor and control.
**Interviewee 12**	The respondent did not provide an answer.

Table S4:

Q4	**How do you assess the legal regulation of the field of illegal trade in wild and especially endangered wildlife species in Slovenia? Are changes needed on legislative or regulatory level?**
**Interviewee 1**	We do not know enough about native species legislation because we focus exclusively on exotic species.
**Interviewee 2**	I think the legislation is adequate, the problem is implementation and enforcement. The number and qualification of hunting, fishing and mountain guards in Slovenia is poor. So is the power.
**Interviewee 3**	We believe that the legal regulations at EU and national level are adequate for CITES species.
**Interviewee 4**	We think that the legal framework is quite clear, but, as we said, changes are needed, especially in the area of detection and prevention, i.e. preventive measures to prevent this type of crime.
**Interviewee 5**	In my opinion: no, it is adequate.
**Interviewee 6**	The legislation is appropriate, but at Slovenian Railways we have our own rules regarding the transport of animals, especially if it does not violate veterinary and other regulations. These rules state that small pets enclosed in cages, baskets or other suitable containers may be transported on passenger trains, provided they can be accommodated as personal luggage. Small dogs may be kept on the lap. A small dog is defined as a dog that does not exceed 30 cm in height at the front, measured from the ground to the withers. Larger dogs must be muzzled and kept on a short leash. Guide dogs for the blind, assistance dogs for the disabled, therapy dogs, and rescue dogs are not required to wear a muzzle and must have visible identification (collar or tag). If other passengers refuse to take animals on the train, the train crew shall decide on the accommodation of passengers and animals. Passengers are obliged to take care of their animals and to compensate the carrier and other passengers for any damage caused by the animals. Dogs may travel in sleeping cars and carriages with beds if the passenger has exclusive use of the compartment. The carriage of small pets in dining cars is not permitted. The only exception is assistance dogs for the blind and disabled. Small pets kept in cages and baskets and small dogs kept on laps are allowed on Intercity (ICS) trains. Larger dogs (over 30 cm) are not allowed on ICS trains. Guide dogs for the blind, disabled assistance dogs, therapy dogs, and rescue dogs are exempt. Larger dogs are charged half the 2nd class fare on passenger trains with no additional surcharges.<br>However, I fully agree that such acts should be regulated and punished with maximum fines and imprisonment. Considering the health problems around the world due to COVID, which I believe are caused by bats, we should think about how to prevent this from happening.
**Interviewee 7**	There are a lot changes that need to be made, there are a lot of grey areas, and last year we actually made a list of all the deficiencies and submitted it.
**Interviewee 8**	The regulations for illegal behavior are adequate, both in terms of felonies and minor offences, and allow for proper implementation of activities and actions by all agencies and institutions involved.
**Interviewee 9**	I am not familiar with the problem of illegal trade and I think that the share of illegal trade in Slovenia is minimal, so I cannot comment on what changes and to what extent would be welcome.
**Interviewee 10**	Legally, trade in endangered species, especially species listed in the appendices of the Convention CITES and in the appendices of the EU Regulation and in all the implementing legislation, is well regulated, but the problem lies in the implementation – monitoring and control of trade, especially through online platforms. There are efforts to regulate the matter at the EU level, but implementation remains a problem in Slovenia, mainly due to the lack of human and financial resources that are urgently needed for this purpose.
**Interviewee 11**	The subject of your questions does not fall within our competence in most cases. We are responsible for monitoring compliance with the conditions for the import and movement of certain wildlife within the European Union. According to Delegated Regulation 2020/688, the movement of wild terrestrial animals between Member States is allowed under certain conditions. Operators can only move wild terrestrial animals from their habitat of origin to a habitat or facility in another Member State if additional requirements are met. A veterinary certificate is issued for each movement of terrestrial animals and each movement is carried out after prior notification. The conditions for movements of wild aquatic animals between Member States to aquaculture facilities are set out in Commission Delegated Regulation 2020/990. The requirements for import of animals into or transit through the Union are laid down in Delegated Regulation 2020/692. In their work, official veterinarians observe practically no unauthorized trade, with the exception of pet animals (mainly dogs). You may come across this issue when processing animal welfare reports. Despite the limited information, we are sending you answers to some of your questions, that we have developed in collaboration with official veterinarians working in this area.

	Because we are not familiar with the comprehensive legislation in this area, it is difficult to answer whether changes should be made at the legislative or regulatory level. The veterinary legislation that applies to domestic animals does not apply to wildlife, or at least not generally, so any action would be very limited and the matter would need careful consideration.
**Interviewee 12**	The hunting organization has a game warden service organized according to the hunting law. Game wardens are officials with professional qualifications appointed by the relevant ministry (Ministry of Agriculture, Forestry and Food). Game wardens supervise only the implementation of the Law on Game and Hunting and have no powers or duties in the area of other regulations, which is a disadvantage. The Slovenian Hunters' Association has been advocating for many years that the game wardens be given the status of nature conservation officers, thus expanding their field of activity. The Slovenian Hunters' Association participates in the project LYNX, which trains hunters and the public in the prevention of illegal lynx hunting and is thus also active in the field of trade prevention. This primarily involves the procedures for detecting and preventing such acts, inspection of found animals and follow-up in cooperation with the police, who have issued a special instruction for this purpose.

Table S5:

Q5	**How do you assess the competence of experts in your field to identify, detect and investigate illegal trade in wildlife and especially endangered wildlife species in Slovenia? Are changes needed in this area, and if so, what are they?**
**Interviewee 1**	I have not heard that there are experts and staff working in this area in Slovenia.
**Interviewee 2**	"There are too few of them, they are not sufficiently trained, and they do not have enough equipment. Maybe setting up an environmental crime investigation group at the level of national investigation agencies would be a step forward - it's just an idea for now.
**Interviewee 3**	We think that the competence of the financial management staff to monitor the implementation of the above regulations is sufficient. We also evaluate as sufficient the cooperation with other competent authorities or the participation of the Financial Administration in the inter-ministerial working group for the relevant area, headed by the Ministry of Environment and within the framework of which the activities of the supervisory authorities are planned and coordinated.
**Interviewee 4**	They are not specifically trained.
**Interviewee 5**	The security personnel of the Port of Koper d.d. are trained to detect this type of illegal trade. If such trafficking is uncovered or detected, the customs administration takes over the procedures in the port area.
**Interviewee 6**	The respondent did not provide an answer.
**Interviewee 7**	In my (perhaps critical) opinion, there is a lack of knowledge and appropriate professionals in this area. Either there are people who have no idea about exotic animals or people who hate such animals and want to ban them. In the absence of legislation that can be effectively invoked, I think it is difficult to do anything about it. I fear that people are again afraid of exotic animals because we, as owners and lovers of these animals, experience discrimination against other animal owners every day. We need to distinguish between illegal hunting of Slovenian reptiles or amphibians (which I admit I have never experienced, but have only read articles about cave animal traps in our underground caves) and the influx of legally bred exotic animals bred by enthusiasts. Cage breeding takes the pressure off for illegal captures and should be encouraged and regulated, but not overly complicated as is currently the case in Slovenia.
**Interviewee 8**	In case of a report or detection of suspected illegal acts related to protected animals and plants, the police examine all information and take all necessary steps and measures based on the law and the profession. Since police officers are not experts in the field of identification of protected animal and plant species, we actively cooperate with the Ministry of Environment and Spatial Planning, the Institute of the Republic of Slovenia for Nature Protection (ZRSVN) and the competent control authority, the Inspectorate for Environment and Spatial Planning, when dealing with criminal cases. In order to raise awareness of this issue and of new trends in crime, the police organize training courses for police officers and criminal investigators, participate in the in education and training of other supervisory authorities, and take part in seminars and conferences abroad.
**Interviewee 9**	No answer was received from the respondent.
**Interviewee 10**	Interministerial cooperation and integration is central to this work. In Slovenia, there is an inter-ministerial working group, which includes the police, customs, supervisory authority and nature protection of the Ministry of Environment, which monitors this area in cooperation with the Institute of the Republic of Slovenia for Nature Protection and specialized organizations and institutions. In order to ensure successful work, it is necessary to organize regular trainings for the monitoring authorities, which were held regularly in previous years, but had to be postponed in recent years due to staff shortages and the pandemic COVID. In addition, in our experience, penalties for offenders in Slovenia are too lenient and the cost of caring for those seized is high. The biggest shortcoming we need to address is the involvement of judicial authorities, especially prosecutors and judges. There is a lack of knowledge, understanding and cooperation at this level both in Slovenia and at the EU level. This has already improved in some EU countries, and the EU Action plan against illegal wildlife trade will be complemented by better integration of this issue, with a focus on good cooperation and integration between EU member states. The Slovenian government has adopted a resolution on the National Program to Prevent and Combat Crime 2019-2023, which also calls for better cooperation with judicial authorities.
**Interviewee 11**	The professional competence of official veterinarians is not at the same level as for pets (most official veterinarians are not trained to handle foreign wildlife species). In addition, procedural law (Inspection Act, General Administrative Procedure Act) severely restricts veterinary officials in the area of illegal trade. The police and the prosecutor's office have sufficient powers.
**Interviewee 12**	Hunters are educated about the prevention of illegal hunting and thus illegal trafficking as a part of the training to obtain a hunting license and a hunting warden's license. This means that this training can be considered part of the prevention of this type of trafficking, which certainly exists in Slovenia. Currently, there are over 20,000 hunters in

	Slovenia, of which more than 850 are sworn game wardens working in all 411 Slovenian hunting families. In the context of game wardens and hunters, it can be said that Slovenian hunters are trained to recognize and identify this type of problem.

Table S6:

**Q6**	**How do you assess the level of awareness among the Slovenian population regarding the competence of experts in your field to identify, detect and investigate illegal trade in wildlife and especially endangered wildlife species in Slovenia and animal welfare? Are changes needed in this area and if so, what are they?**
**Interviewee 1**	Based on our workshops and exhibitions that we organize annually; we see that awareness of the issue is increasing. Of course, we focus on exotic animals and environmental protection, but we also always include Slovenian reptiles and teach the participants about the proper treatment of the environment and native animals. The public is becoming more sensitive day by day
**Interviewee 2**	Slovenians have always been very aware and knew very well which animals and plants are protected. The problem arises when profit takes over. Especially when people are struggling to survive. For example, killing a bear can fill a person's refrigerator for some time, or an illegal hunter can pay someone to show him a bear so he can live off that money.
**Interviewee 3**	In our opinion, the awareness of the Slovenian population regarding the protection of CITES wildlife species has improved in recent years, which is also reflected in the statistics on confiscations. In our opinion, the financial administration has also contributed to this awareness, as the issue of illegal trade in protected wildlife species is regularly presented at various events we organize for the public. The specimens seized by Slovenian customs authorities in recent years, which we also keep for educational purposes, are also displayed at Jože Pučnik Ljubljana Airport to inform and raise awareness among travelers.
**Interviewee 4**	In our estimation, awareness of the identification, detection and investigation of illicit trafficking is low, both among the general public and among professionals. In any case, we believe that it is necessary to monitor the development of illicit trafficking in cooperation with foreign authorities and adjust the level of risk, and on this basis, increase the level of training, identification, detection and investigation of illicit trafficking.
**Interviewee 5**	I think the population is sufficiently aware.
**Interviewee 6**	No answer was received from the respondent.
**Interviewee 7**	No answer was received from the respondent.
**Interviewee 8**	The police and other authorities confronted with the trade of protected animal and plant species try to contribute to greater and better public awareness through their activities. The police are mostly involved in the creation of prevention materials or in press conferences, we also participate in international (prevention) campaigns and inform the public through the media about successfully concluded cases.
**Interviewee 9**	No answer was received from the respondent.
**Interviewee 10**	Representatives of the Ministry of the Environment (formerly Slovenian Environment Agency) regularly give lectures at the Biotechnical Faculty on protected and endangered wildlife and CITES. Otherwise, the Ministry of Environment draws attention to the issue every year on the occasion of World Wildlife Day (March 3rd). The issue is increasingly covered in the media, and the public reacts in different ways.
**Interviewee 11**	In terms of public awareness and sensitivity, we think they are good thanks to the media.
**Interviewee 12**	In our estimation, the awareness of the Slovenian population is at a satisfactory level, as this problem is not urgent. However, this is certainly not a reason not to carry out further awareness-raising activities on this issue. However, it is true that not enough attention is paid to this issue. Illegal trafficking crimes is mostly reported in the media when the police uncover such crimes during border controls and surveillance in transit areas. The existing rules for listing endangered plant and animal species in the Red List are apparently not very well known.
